# Teriparatide Associated with Fewer Refractures and Higher Body Heights of Cemented Vertebrae after Vertebroplasty: A Matched Cohort Study

**DOI:** 10.1038/s41598-020-62869-0

**Published:** 2020-04-07

**Authors:** Yi-Shan Yang, Yi-Syue Tsou, Wen-Cheng Lo, Yung-Hsiao Chiang, Jiann-Her Lin

**Affiliations:** 1 Department of Neurosurgery, Taipei Medical University Hospital, Taipei, Taiwan; 2 Division of Neurosurgery, Department of Surgery, School of Medicine, College of Medicine, Taipei Medical University, Taipei 11031, Taiwan; 3 Taipei Neuroscience Institute, Taipei Medical University, Taipei, Taiwan

**Keywords:** Neurological disorders, Fracture repair

## Abstract

Refracture of cemented vertebrae occurs commonly after vertebroplasty (VP) for osteoporotic vertebral compression fracture (OVCF). It can result in severe pain or neurological deficit, but no preventive medication is available. Owing to the bone anabolic benefits of teriparatide (TP), this study was aimed to compare the outcomes of cemented vertebrae with TP to those without TP. Patients who received VP for OVCF with at least 1 year follow-up were included. The anterior body height (ABH) and middle body height (MBH) and kyphotic angle (KA) were measured before VP and 1 week and at least 1 year after VP. Refracture was defined as a 15% decrease in ABH or MBH and 8° decrease in KA compared with those at postoperative 1 week. The clinical outcomes were evaluated. 35 VP procedures in 21 patients treated with TP (TP group), and, matched to that, 29 out of 133 patients treated with VP alone (VP group) were included. One year after VP, ABH and MBH were significantly greater, except KA, in the TP group (VP group vs. TP group: KA − 4.97° ± 12.1 vs. −2.85° ± 12.21°, *p* = 0.462, ABH 1.56 ± 0.48 cm vs. 1.84 ± 0.56 cm, *p* = 0.027, MBH 1.49 ± 0.39 cm vs. 1.73 ± 0.41 cm, *p* = 0.017). The refracture rates of KA, ABH, and MBH were significantly lower in the TP group (VP group vs. TP group: KA 42.11% vs.8.57%, *p* < 0.001; ABH 76.32% vs. 28.57%, *p* < 0.0001; MBH 76.32% vs. 28.57%, *p* < 0.0001). In single-level subgroup comparison, TP was associated with better improvement of pain VAS and better radiological outcomes. TP was associated with higher BHs and fewer refractures than VP alone, with comparable clinical outcomes 1 year after VP. TP may be associated with better improvement of pain VAS in those with single-level VP procedure. Higher BH was due to the better maintenance effect of TP.

## Introduction

Refracture of cemented vertebrae after vertebroplasty (VP) occurs frequently in patients with osteoporotic vertebral compression fracture (OVCF). Different studies have reported different incidences of refracture^1–6^. Refracture incidence ranged from 0.56% to 76% depending on the definition and follow-up period. Some refractures resulted in severe pain, instability, and even neurological deficits requiring further interventions^2,7–9^. Kyphoplasty (KP) with an intravertebral reduction device (IRD) has been reported as a solution to prevent refracture with stronger anterior mechanical support^6^. However, after VP, no medical solution exists so far.

Teriparatide (TP) is the recombinant human parathyroid hormone (1–34) that increases bone mass^10,11^ and decreases the risk of new vertebral fracture in patients with osteoporosis^12,13^. Its antiosteoporosis effects depend on the enhancement of osteoblast formation^14^ and prevention of osteoblast apoptosis^15^. Owing to the anabolic effect, TP had been used to promote the process of bone healing after fracture^11,16–28^. For fractures without internal implant fixation, TP was beneficial for nonweight-bearing regions^22,28^ but not for weight-bearing regions^25^. The role of TP is very controversial for fractures in weight-bearing regions. TP did not demonstrate benefits for enhancing bone-implant interface strength^17^ and the fusion rates^20,23^, although it was associated with better clinical outcomes^16,20,23^. For OVCF without VP, TP was associated with higher body heights (BHs)^29^ and pain reduction^26,30^. However, the local bone environment inside the fractured vertebrae was more complicated by cement after VP^31^. Hence, an interesting question arises: can TP help maintain the BH and prevent refracture under such a complicated bone environment in OCVF after VP?

This study assessed whether TP can reduce refracture risk in OVCF patients after VP by comparing the radiological and clinical outcomes of cemented vertebrae with TP with those without TP.

## Material and methods

### Patient selection

This retrospective case-matched study was approved by the Taipei Medical University Joint Institutional Review Board (TNU-JIRB N201705068) and informed consent was waived. All methods were performed in accordance with the relevant guidelines and regulations. For 546 patients, there were 660 operative records about VP-treated thoracic and lumbar OVCFs from January 2013 to December 2016 in Taipei Medical University Hospital. We included patients who underwent VP with or without TP before October 31, 2016 and had at least 1 year of follow-up. Patients who were treated with KP; were followed up for less than 1 year; had other surgical interventions; exhibited neurological deficits; were diagnosed with neoplastic spinal cord compression; had unmanageable bleeding disorders; had systemic or local spinal infections; had severe comorbidities of the heart, liver, kidney, or lung with intolerance to surgery; or had no available preoperative magnetic resonance imaging (MRI) scans were excluded. After reviewing the aforementioned criteria, we included 239 levels of OVCF in 166 patients who received VP. There were 167 OVCFs in 133 patients without TP and 35 OVCFs in 21 patients with TP (TP group). TP was started during the duration 1 month before VP to 6 weeks after VP and used continually for at least 3 months. TP was subcutaneously administrated daily based on the recommended protocol (20 microgram daily)^12,13^. According to the guideline suggested by the Taiwan National Health Insurance, teriparatide is indicated when patients have all of the followings:

1. Bone marrow density (BMD) < −3.0
2. More than one vertebral or hip fractures
3. Not tolerable to other antiosteoporotic therapy

**Figure 1:**
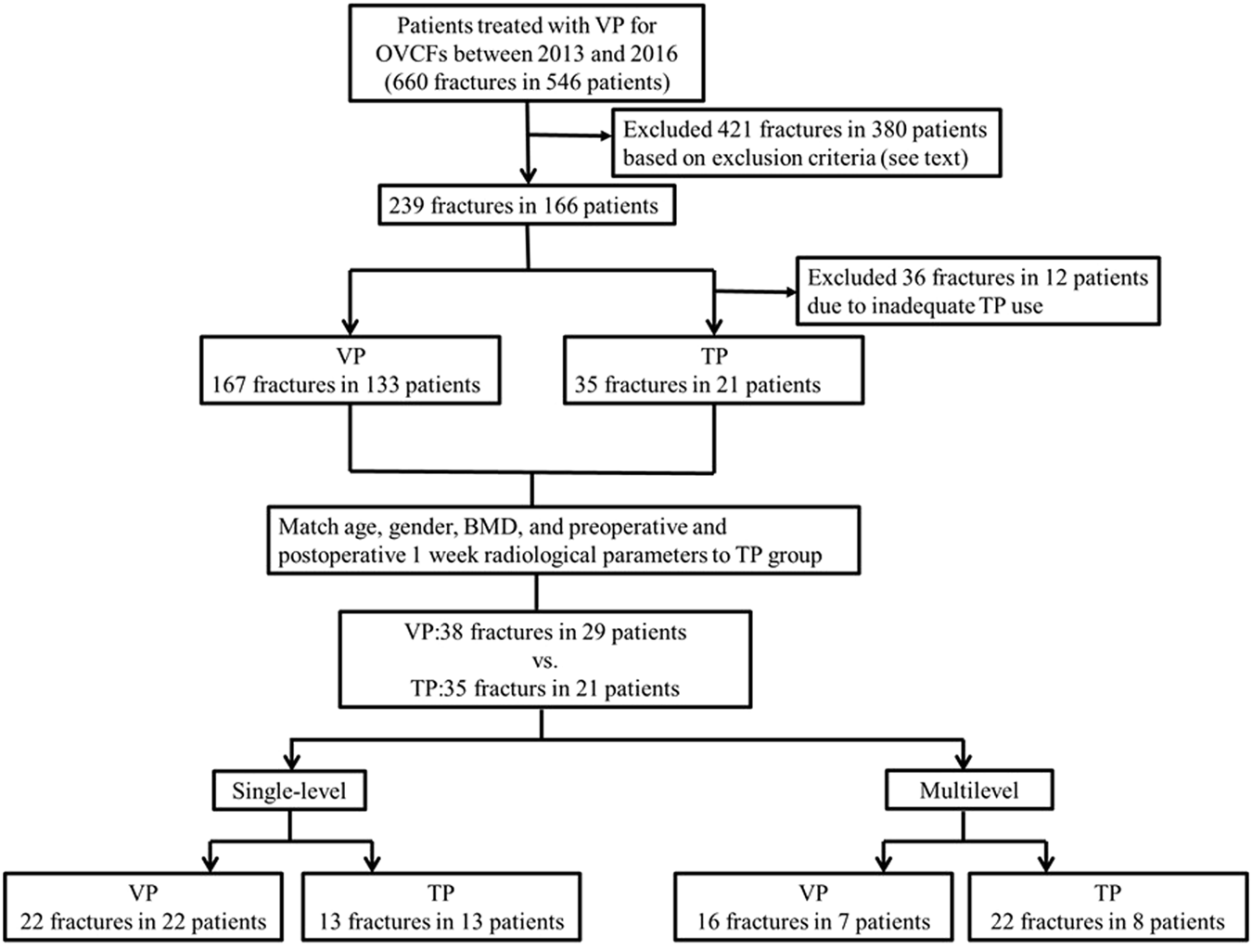
Flowchart of the eligible subjects (BMD: bone marrow density, OVCF: osteoporotic vertebral compression fracture, TP: teriparatide, VP: vertebroplasty).

In addition, TP was also suggested for the patients if they have one of the following indications: 1. Glucocorticoid-induced osteoporosis patients with fragile fracture, 2. More than 2 vertebral fractures simultaneously, 3. Patients with subacute sequential vertebral fracture following the first vertebral fracture (less than 3 months). OVCF was detected through MRI, with bone edema in the fractured vertebra on T2-weighted short tau inversion recovery sequences or vertebral body enhancement on MRI-contrasted T1-weighted sequences. We further matched VP patients to TP patients by age, BMD, gender, preoperative and postoperative 1 week radiological parameters (the kyphotic angle (KA), anterior body height (ABH), and middle body height (MBH)). Consequently, there were 38 levels of OVCF in 29 patients without TP (VP group) and 35 levels of OVCF in 21 patients with TP. Among them, 7 out of 29 patients in VP group and 8 out of 21 in TP group had multilevel VP procedures. Then we did the subgroup analysis of single-level and multilevel VP procedures respectively (Fig. 1).

### Radiological outcomes

The radiological parameters were measured on the lateral lumbar spine flexion and extension X-ray obtained when the patients were lying. The average of measurement of flexion and extension X-ray was included into the subsequent analysis. Two experience neurosurgeons independently did the radiological measurement and they were blinded to the clinical information of the patients. The intraclass correlation coefficient was conducted to test the inter-rater reliability. The intraclass correlation coefficient showed that the reliability between these 2 raters were good to excellent in total 267 radiological measurements for KA, ABH, and MBH (KA: 0.989, ABH: 0.90; MBH: 0.888, respectively) (Supplement Table 2).

**Figure 2:**
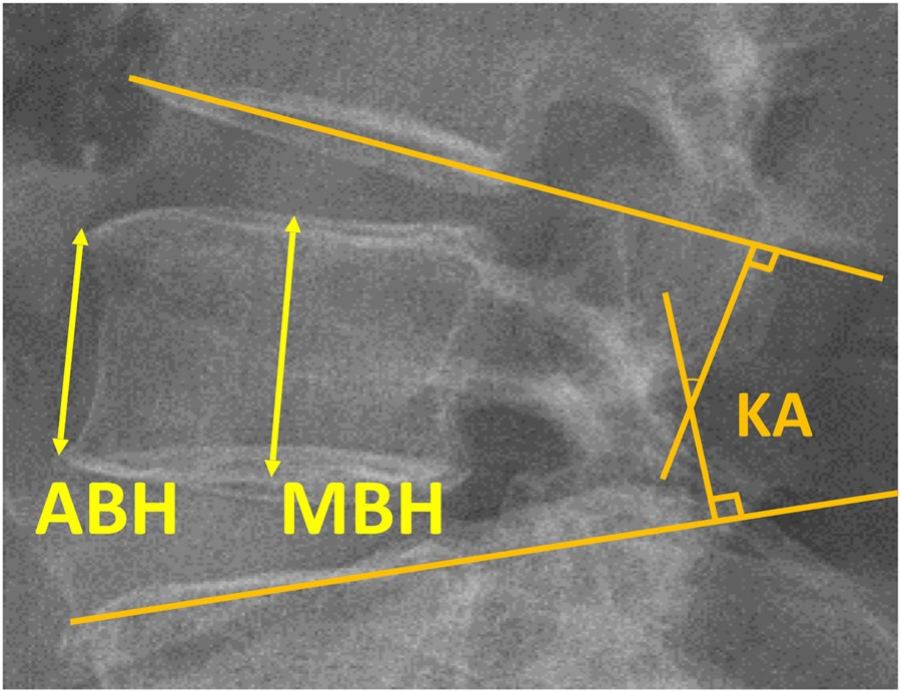
The measurements of radiological outcomes (ABH: anterior body height, MBH: middle body height, KA: kyphotic angle).

The KA, ABH, and MBH were measured before VP (preop), 1 week after VP (postop-1w), and 1 year after VP (postop-1y). Radiological parameters of the cemented vertebrae usually did not change 6 months after VP^6^. KA was measured from the inferior end plate of the vertebral body, which was above one level of the injured vertebral body, to the superior end plate of the vertebral body, which was below one level of the injured vertebral body (Fig. 2). ABH and MBH were defined as the distance between the upper and lower edges at the anterior and middle, respectively, of the vertebral body (Fig. 2).

To investigate the maintenance of body heights and kyphotic angles, we evaluated the changes of radiological parameters after VP. The maintenance rate (MR) of the vertebral BH were calculated using the following formulae: MR of the vertebral BH = postop-1y BH/postop-1w BH. Furthermore, the difference of the KA (DKA) were calculated using the following formulae: DKA = postop-1y KA − postop-1w KA. MR or DKA indicated the maintenance of BH or KA of the cemented vertebrae after VP. Refracture was defined as a 15% decrease in ABH or MBH (MR < 0.85) and 8° decrease in KA (DKA > 8°) compared with those at postoperative 1 week. MR or DKA indicated the maintenance of BH or KA of the cemented vertebrae after immediate reduction by VP.

### Clinical outcomes

Patient-reported outcomes of the case-matched VP and TP groups were evaluated preoperatively and more than 1 year postoperatively. The preoperative visual analogue scale (VAS) and Oswestry disability index (ODI; Chinese version)^32^ were recorded by reviewing charts, and the postoperative outcomes were obtained through phone interviews.

### Statistical analysis

These results are presented as the mean ± standard deviation. We used Prsim 8 for conducting statistical analysis and SAS 9.4 to match the patients between the VP and TP groups. Student’s unpaired two-tailed t-test was used for comparing the radiological outcomes of the two groups at each time point. Mann-Whitney test was used for ranking parameters. The chi-squared test was used for comparing noncontinuous parameters. Intraclass correlation coefficient estimates and their 95% confident intervals were calculated using SPSS statistical package version 20 (SPSS Inc, Chicago, IL) based on a single-rating, consistency, 2-way mixed effects model^33^.

## Results

### Comparison of TP and VP group

A total of 167 OVCFs in 133 patients were treated with VP without TP (VP group), and 35 OVCFs in 21 patients were treated with VP and TP (TP group). No significant differences in age, gender, fracture level, and body mass index (BMI) were observed between the TP and total VP groups, except BMD (TP vs. VP: −2.68 ± 0.98 vs. −1.9 ± 1.32, *p* < 0.01) (Supplement Table 1). The comparison of radiological outcomes between the TP group and the all VP patients (n = 113) was shown in the supplement material (Supplement Fig. 1). After we matched the VP group to the TP group, there were 38 OVCFs in 29 patients (VP group). Furthermore, no differences in age, gender, fracture level, BMI, BMD, ODI, preoperative and postoperative-1-week radiological parameters were observed between the TP and VP groups, but preoperative pain VAS was reported more intense in TP group (Table 1).

**Table 1:** Comparison of VP and TP group.

	VP	TP	P-Value
n	29	21	
Fractures	38	35	
Multilevel	7	8	
**Segment**			
1	22	13	
2	6	4	
3	0	3	
4	1	0	
5	0	1	
**Age**	78.72 ± 7.42	79.19 ± 7.08	0.789
**Gender**			
F	23	17	>0.9999
M	6	4	
**BMI**			
	22.96 ± 3.42	23.14 ± 4.46	0.878
**BMD**			
	−2.52 ± 1	−2.68 ± 0.98	0.565
**ODI**			
Preop	65.73 ± 8.81	71.47 ± 9.67	0.101
Postop-1y	21.13 ± 18.5	33.87 ± 16.24	0.079
**VAS**			
Preop	7.5 ± 1.83	8.57 ± 1.88	0.048*
Postop-1y	2.69 ± 2.36	2.33 ± 2.35	0.722
Pain VAS improvement	4.27 ± 3.01	6.23 ± 2.99	0.084
**KA**			
Preop	−3.18 ± 10.65	−4.77 ± 12.74	0.572
Postop-1w	0.56 ± 10.42	−0.71 ± 11.9	0.891
Postop-1y	−4.97 ± 12.05	−2.85 ± 12.21	0.462
**DKA**			
Postop-1y	−5.59 ± 7.31	−2.14 ± 4.16	0.008**
**ABH**			
Preop	1.58 ± 0.61	1.75 ± 0.62	0.249
Postop-1w	1.98 ± 0.53	2.06 ± 0.55	0.289
Postop-1y	1.56 ± 0.48	1.84 ± 0.56	0.027*
**ABHMR**			
Postop-1y	0.79 ± 0.11	0.89 ± 0.09	<0.0001****
**MBH**			
Preop	1.61 ± 0.55	1.63 ± 0.49	0.88
Postop-1w	1.88 ± 0.48	1.94 ± 0.43	0.574
Postop-1y	1.49 ± 0.39	1.73 ± 0.41	0.017*
**MBHMR**			
Postop-1y	0.8 ± 0.09	0.89 ± 0.09	<0.0001****

ABH: anterior body height; BMD: bone marrow density; BMI: body mass index; KA: kyphotic angle; MBH: middle body height; MR: maintenance ratio; ODI: Oswestry disability index; Preop: preoperative; Postop: postoperative; TP: teriparatide; VP: vertebroplasty.

### Radiological outcomes

**Figure 3:**
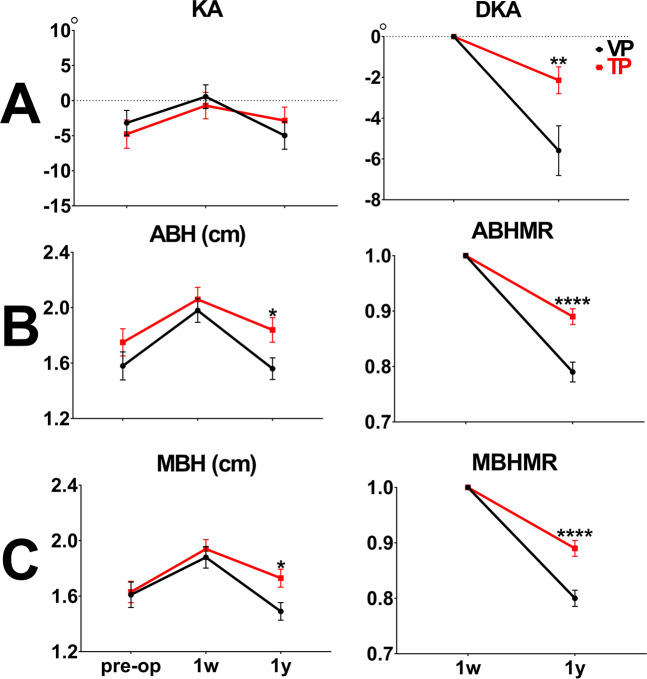
A comparison of radiological outcomes between the VP and TP groups at preop, postop-1w, and postop-1y (ABH: anterior body height, KA: kyphotic angle, DKA: difference of kyphotic angle, MBH: middle body height, MR: maintenance rate, TP: teriparatide, VP: vertebroplasty, 1w: post-operative 1 week, 1 y: post-operative 1 year, *p < 0.05, **p < 0.01, ***p < 0.001, ****p < 0.0001).

First, the radiological outcomes of the TP group were compared with those of the VP group (Table 1). No difference in postop-1y KA was observed in either group (VP group vs. TP group: −4.97° ± 12.05° vs. −2.85° ± 12.21°, *p* = 0.462). DKA was more efficient in the TP group than in the VP group (VP group vs. TP group: −5.59° ± 7.31° vs. −2.14° ± 4.16°, *p* = 0.008) (Fig. 3A). Postop-1y ABH was significantly higher in the TP group than in the VP group (VP group vs. TP group: 1.56 ± 0.48 cm vs. 1.84 ± 0.56 cm, *p* < 0.01). ABHMR was significantly more efficient in the TP group (VP group vs. TP group: 0.79 ± 0.11 vs. 0.89 ± 0.09, *p* < 0.0001) (Fig. 3B). Postop-1y MBH was significantly higher in the TP group than in the VP group (VP group vs. TP group: MBH 1.49 ± 0.39 cm vs. 1.73 ± 0.41 cm, *p* = 0.017). MBHMR was significantly more efficient in the TP group (VP group vs. TP group: 0.80 ± 0.09 vs. 0.89 ± 0.09, *p* < 0.0001) (Fig. 3C). Furthermore, the refracture rates of KA, ABH, and MBH were significantly lower in the TP group compared with the VP group 1 year after VP (VP group vs. TP group: KA 42.11% vs. 8.57%, *p* = 0.001; ABH 76.32% vs. 28.57%, *p* < 0.0001; MBH 76.32% vs. 28.57%, *p* < 0.0001) (Table 2).

**Table 2:** Refracture rates of VP and TP groups.

	TP	VP	P-Value
KA	8.57%	42.11%	0.001**
ABH	28.57%	76.32%	<0.0001****
MBH	28.57%	76.32%	<0.0001****

ABH: anterior body height; KA: kyphotic angle; MBH: middle body height; TP: teriparatide; VP: vertebroplasty.

### Clinical outcomes

Preoperative pain VAS and ODI were significantly improved in both the TP and VP groups. No significant difference was observed between the two groups in terms of postop-1y pain VAS and its improvement and ODI (Table 2).

### Single-level subgroup analysis

In the comparison of single-level VP and TP groups, there was no differences in age, gender, BMI, BMD, preoperative ODI, preoperative pain VAS, preoperative and postoperative 1 week radiological parameters were observed between the TP and VP groups, except preoperative ABH of TP group was significantly greater (Table 3).

**Table 3:** Comparison of Single-level VP and TP group.

	VP	TP	P-Value
n	22	13	
Fractures	22	13	
Age	79.23 ± 7.65	79.08 ± 8.59	0.913
**Gender**			
F	16	11	0.68
M	6	2	
**BMI**			
	23.07 ± 3.68	24 ± 4.89	0.526
**BMD**			
	−2.27 ± 0.96	−2.57 ± 0.93	0.364
**ODI**			
Preop	64.4 ± 8.78	73.56 ± 11.04	0.06
Postop-1y	21.27 ± 19.8	30.22 ± 19.3	0.376
**VAS**			
Preop	7.27 ± 1.9	8.33 ± 2.18	0.084
Postop-1y	3 ± 2.53	1.11 ± 1.69	0.082
Pain VAS improvement	4.27 ± 2.72	7.22 ± 2.77	0.028*
**KA**			
Preop	−3.75 ± 10.9	−4.05 ± 12.35	0.942
Postop-1w	−1.46 ± 9.7	0.24 ± 12.01	0.656
Postop-1y	−7.71 ± 10.7	−2.18 ± 12.3	0.18
**DKA**			
Postop-1y	−5.42 ± 6.49	−2.42 ± 5.05	0.175
**ABH**			
Preop	1.49 ± 0.54	1.97 ± 0.57	0.021*
Postop-1w	1.9 ± 0.44	2.21 ± 0.47	0.072
Postop-1y	1.52 ± 0.42	1.95 ± 0.5	0.011*
**ABHMR**			
Postop-1y	0.79 ± 0.09	0.89 ± 0.09	0.011*
**MBH**			
Preop	1.58 ± 0.47	1.77 ± 0.48	0.266
Postop-1w	1.84 ± 0.39	2.03 ± 0.43	0.144
Postop-1y	1.48 ± 0.35	1.8 ± 0.38	0.019*
**MBHMR**			
Postop-1y	0.81 ± 0.07	0.89 ± 0.06	0.003**

ABH: anterior body height; BMD: bone marrow density; BMI: body mass index; KA: kyphotic angle; MBH: middle body height; MR: maintenance ratio; ODI: Oswestry disability index; Pre-op: preoperative; Post-op: postoperative; TP: teriparatide; VP: vertebroplasty.

### Radiological outcomes

**Figure 4:**
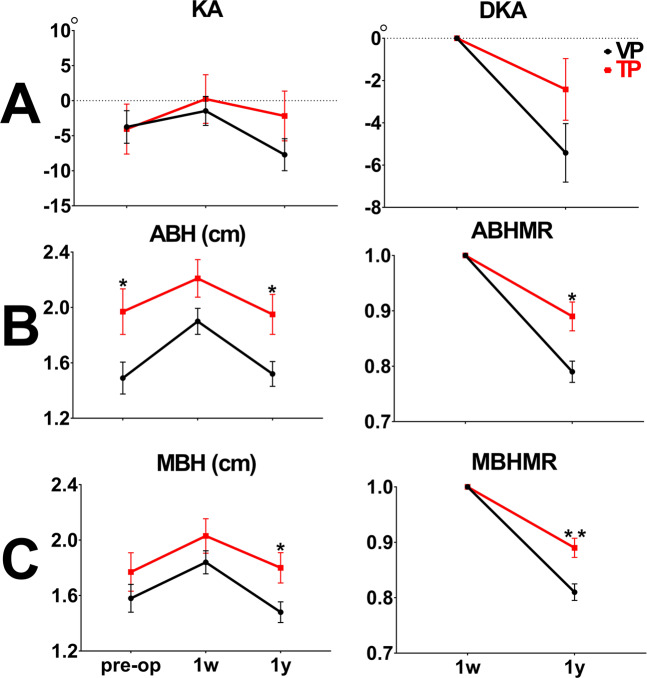
A comparison of radiological outcomes between the single-level VP and TP groups at preop, postop-1w, and postop-1y (ABH: anterior body height, KA: kyphotic angle, DKA: difference of kyphotic angle, MBH: middle body height, MR: maintenance rate, TP: teriparatide, VP: vertebroplasty, 1w: post-operative 1 week, 1 y: post-operative 1 year, *p < 0.05, **p < 0.01, ***p < 0.001, ****p < 0.0001).

No difference in postop-1y KA and DAK was observed in either group (Single-level VP group vs. TP group: postop-1y KA − 7.71° ± 10.7° vs. −2.18° ± 12.3°, *p* = 0.18; DKA − 5.42° ± 6.49° vs. −2.42° ± 5.05°, *p* = 0.175). (Fig. 4A). Postop-1y ABH was significantly higher in the TP group than in the VP group (Single-level VP group vs. TP group: 1.52 ± 0.42 cm vs. 1.95 ± 0.5 cm, *p* = 0.011). ABHMR was significantly more efficient in the TP group (Single-level VP group vs. TP group: 0.79 ± 0.09 vs. 0.89 ± 0.09, *p* = 0.011) (Fig. 4B). Postop-1y MBH was significantly higher in the TP group than in the VP group (Single-level VP group vs. TP group: MBH 1.48 ± 0.35 cm vs. 1.8 ± 0.38 cm, *p* = 0.019). MBHMR was significantly more efficient in the TP group (Single-level VP group vs. TP group: 0.81 ± 0.07 vs. 0.89 ± 0.06, *p* = 0.003) (Fig. 4C).

### Clinical outcomes

Pain VAS improvement in TP group was significant greater (Single-level VP group vs. TP group: 4.27 ± 2.72 vs. 7.22 ± 2.77, *p* = 0.028). No significant difference was observed between the two groups in terms of postop-1y pain VAS and ODI, (Table 3).

### Multilevel subgroup analysis

In the comparison of multilevel VP and TP groups, there was no differences in age, gender, BMI, BMD, preoperative ODI, preoperative pain VAS, preoperative and postoperative 1-week radiological parameters were observed between the TP and VP groups. However, there were more patients who had fractures at the same time in VP group than in TP group (Multilevel VP vs. TP group: 5 (83%) vs. 1 (14%), p = 0.041) (Table 4).

**Table 4:** Comparison of multilevel VP and TP group.

	VP	TP	P-Value
n	7	8	
Fractures	16	22	
**Segment**			
2	6	4	
3	0	3	
4	1	0	
5	0	1	
Adjacent segment	3 (42.86%)	7 (86.67)	0.123
Fractures at the same time	5 (83%)	1 (14%)	0.041*
Age	77.14 ± 6.91	79.38 ± 4.07	0.452
**Gender**			
F	7	6	0.467
M	0	2	
**BMI**			
	22.64 ± 2.66	21.73 ± 3.49	0.585
**BMD**			
	−3.3 ± 0.74	−2.86 ± 1.09	0.387
**ODI**			
Preop	68.4 ± 9.21	68.33 ± 6.86	0.989
Postop-1y	20.8 ± 17.2	39.33 ± 9.09	0.048*
**VAS**			
Preop	8.33 ± 1.53	8.92 ± 1.43	0.589
Postop-1y	2 ± 2	4.17 ± 2.04	0.048*
Pain VAS improvement	4.25 ± 4.19	4.75 ± 2.89	0.828
**KA**			
Preop	−2.27 ± 10.7	−5.16 ± 13.22	0.498
Postop-1w	1.97 ± 10.1	−1.23 ± 12.09	0.415
Postop-1y	−2.82 ± 12.9	−3.22 ± 12.44	0.927
**DKA**			
Postop-1y	−5.94 ± 8.84	−1.98 ± 3.71	0.071
**ABH**			
Preop	1.72 ± 0.69	1.63 ± 0.63	0.692
Postop-1w	2.04 ± 0.59	1.98 ± 0.59	0.769
Postop-1y	1.58 ± 0.54	1.78 ± 0.59	0.324
**ABHMR**			
Postop-1y	0.77 ± 0.14	0.89 ± 0.09	0.005**
**MBH**			
Preop	1.66 ± 0.67	1.55 ± 0.5	0.578
Postop-1w	1.89 ± 0.52	1.89 ± 0.44	0.992
Postop-1y	1.48 ± 0.42	1.69 ± 0.43	0.171
**MBHMR**			
Postop-1y	0.79 ± 0.12	0.89 ± 0.11	0.015*

ABH: anterior body height; BMD: bone marrow density; BMI: body mass index; KA: kyphotic angle; MBH: middle body height; MR: maintenance ratio; ODI: Oswestry disability index; Pre-op: preoperative; Post-op: postoperative; TP: teriparatide; VP: vertebroplasty.

### Radiological outcomes

**Figure 5:**
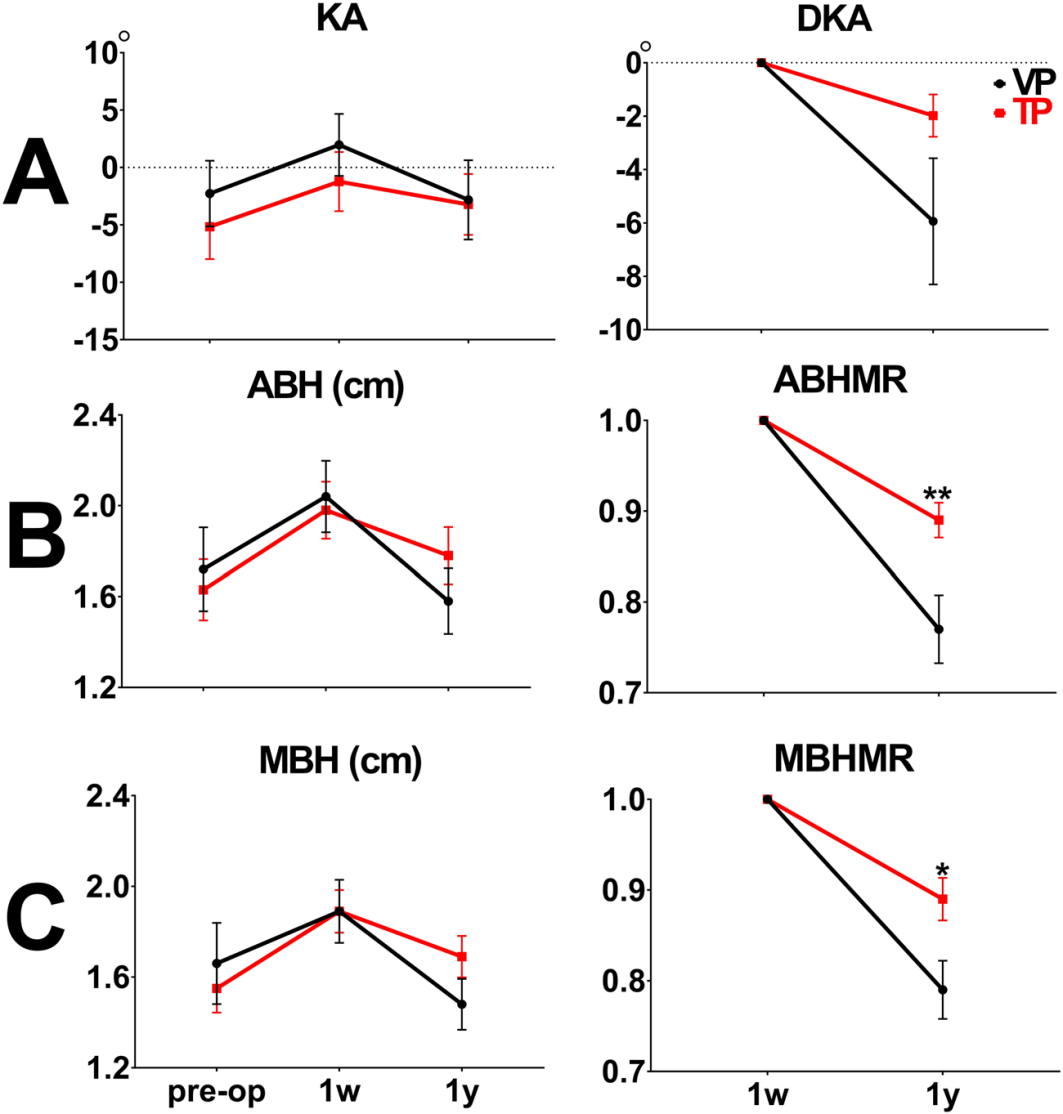
A comparison of radiological outcomes between the multilevel VP and TP groups at preop, postop-1w, and postop-1y (ABH: anterior body height, KA: kyphotic angle, DKA: difference of kyphotic angle, MBH: middle body height, MR: maintenance rate, TP: teriparatide, VP: vertebroplasty, 1w: post-operative 1 week, 1 y: post-operative 1 year, *p < 0.05, **p < 0.01, ***p < 0.001, ****p < 0.0001).

No difference in postop-1y KA and DAK was observed in either group (Multilevel VP group vs. TP group: postop-1y KA −2.82° ± 12.9° vs. −1.98° ± 12.44°, *p* = 0.927; DKA − 5.94° ± 8.84° vs. −1.98° ± 3.71°, *p* = 0.071). (Fig. 5A). Postop-1y ABH was not different between two groups (Multilevel VP group vs. TP group: 1.58 ± 0.54 cm vs. 1.78 ± 0.59 cm, *p* = 0.324). ABHMR was significantly more efficient in the TP group (Multilevel VP group vs. TP group: 0.77 ± 0.14 vs. 0.89 ± 0.09, *p* = 0.005) (Fig. 5B). Postop-1y MBH was not different between two groups (Multilevel VP group vs. TP group: MBH 1.48 ± 0.42 cm vs. 1.69 ± 0.43 cm, *p* = 0.171). MBHMR was significantly more efficient in the TP group (Multilevel VP group vs. TP group: 0.79 ± 0.12 vs. 0.89 ± 0.11, *p* = 0.015) (Fig. 5C).

### Clinical outcomes

Postop-1y pain VAS and ODI in TP group was significant greater (Multilevel VP group vs. TP group: VAS 2 ± 2 vs. 4.17 ± 2.04, *p* = 0.048; ODI 20.8 ± 17.2 vs. 39.33 ± 9.09, *p* = 0.048). (Table 4).

## Discussion

Based on this study, OVCF patients in the TP group had significantly lower refracture rates than those in the VP group 1 year postoperatively. ABH, MBH, and KA were all favorable in the TP group. TP was associated with better maintenance effects in ABH, MBH, and KA. The pain VAS and ODI were significantly reduced 1 year after VP in both the groups. Most importantly, TP was associated with better improvement of pain VAS in those with single-level VP procedure. The beneficial effects of TP for VP-treated OVCFs were first demonstrated in our study.

Our study showed that TP may reduce the risk of refracture in cemented vertebrae after VP for OVCFs. The risk factors for refracture comprise inherent and procedure-related risk factors. The inherent risk factors are low BMD^1^ (T score < −2.2), old age^1^, loss of preoperative ABH^1^, a history of other fractures^34^, a greater local KA and a greater sagittal index^35^, and glucocorticoid-induced osteoporosis^36,37^. The procedure-related risk factors are receiving KP^38^, a lower volume of injected cement^38^, significant ABH restoration^2,4,5^, and solid lump filling cement^2^. Moreover, vertebral bone marrow integrity assessed through quantitative preprocedural MRI is associated with refracture^3^. Although many risk factors have been identified in previous studies, there is little information about refracture prevention. In our previous study, KP using IRD was demonstrated to be associated with significantly lower refracture rates than VP alone^6^. In addition, the current study demonstrated that TP was also a preventive factor for refracture.

Our study first demonstrated that TP was still beneficial in a fractured and cemented bony environment. TP was used for not only increasing BMD but also enhancing bone healing. Li N. *et al*.^39^ proved that TP boosts early-stage fracture healing by upregulating the levels of osteogenesis-specific Runx2 mRNA and protein expression in a rat model. Lin *et al*.^40^ presented that TP increased the union rate in a mouse atrophic nonunion model through cortical bridging of the fracture gap with mature bone. In an animal model and clinical data^41^, TP improved osteointegration of implant through the thickening of bone trabeculae and increased bone mass in the peri-implant area. However, no study has assessed whether TP could reduce the risk of refracture of cemented vertebrae. The local bone environment inside the fractured vertebrae is complicated by cement, and therefore, bone healing is compromised^31^. A histological study of the human vertebra revealed the presence of necrotic bone tissue, foreign body giant cells, and macrophages in the fibrous membrane surrounding cement^31^. These inflammatory changes inside the cemented vertebrae may hinder bone healing^42^. In our study, TP was significantly associated with greater BH and KA, suggesting that TP enhances bone formation even in a fractured, inflammatory, and cemented bony environment.

Our current study demonstrated that BH and KA were restored immediately after VP to a certain extent, and TP maintained the BH and KA that were restored through VP after 1 year. Our previous study indicated that BH and KA improved immediately after VP but decreased gradually after 6 months^6^. The immediate post-VP improvement in BH and KA resulted from the positional reduction during VP, but their restorations disappeared gradually 6 months later. To evaluate the maintenance after VP, MR was calculated by comparing BH and KA to those at immediate post-VP period. BH was significantly higher in the TP group 1 year after VP than in the VP group (Figs. 3 and 4). The postop-1y improvement in ABH or MBH in the TP group was due to better MR. BH was significantly improved with TP through long-term maintenance and not by immediate restoration. By contrast, KP with IRD led to significantly greater improvement in BH through immediate restoration^6^. Postop-1y KA in the TP group was greater although no significance was observed. TP was associated with better KA maintenance, suggesting its long-term maintenance effect.

The benefit of TP in clinical outcome was not evident in the whole group comparison, but the subgroup analysis revealed that TP was associated with the better pain VAS improvement in single-level group. Although these two groups were matched for some characteristics, they were different in characteristics of multiple fractures. Thereafter, when only single-level group were compared, the benefit of TP was evident in this simple condition. However, when multilevel group were compared, the multilevel TP group had worse clinical outcome than the multilevel VP group. It should not be interpreted as the adverse effect of TP on clinical outcomes because patients in TP group tended to have worse clinical conditions because of the indications for TP. In this retrospective study, TP was used for the patients with severe osteoporosis or those with more tendency to have a subsequent fracture. Accordingly, patients in multilevel TP group had more fractures. In multilevel TP group, 4 out of 8 patients had> or = 3 level fractures while only 1 of 7 patients in multilevel VP group had> or = 3 level fractures. Patients with more fractures tended to report worse clinical outcomes. In addition, 7 (86%) patients of multilevel TP group suffered from fractures at distant time while 2 (17%) of multilevel VP group suffered from fractures at distant time (Table 4). Actually, those 7 patients of multilevel TP gorup  suffered from a subsequent fracture less than 3 months after the first fracture. Repeated fractures in a short duration may make the clinical outcomes worse, since the adverse effects of the first fracture do not cease yet. A study with larger sample size and the same selection criteria for both TP and VP group is needed to elucidate the benefit of TP in the future.

This study had some limitations. Data in this retrospective study were obtained from a single medical hospital, and the study had a relatively small sample size along with a relatively short follow-up duration of 1 year. The selection criteria of surgical procedures varied among the surgeons in this study. Therefore, a long-term, prospective multicenter study enrolling a large sample size with a favorable follow-up rate is warranted.

## Conclusion

TP after VP was associated with higher BH and fewer refractures than VP alone, with comparable clinical outcomes 1 year after VP. TP may be associated with better improvement of pain VAS in those with single-level VP procedure. Higher BH was due to the more efficient maintenance effect that was associated with TP.

### Supplementary information

Supplementary table 1.
