# Author Correction: Teriparatide Associated with Fewer Refractures and Higher Body Heights of Cemented Vertebrae after Vertebroplasty: A Matched Cohort Study

**DOI:** 10.1038/s41598-024-60531-7

**Published:** 2024-04-29

**Authors:** Yi-Shan Yang, Yi-Syue Tsou, Wen-Cheng Lo, Yung-Hsiao Chiang, Jiann-Her Lin

**Affiliations:** 1 Department of Neurosurgery, Taipei Medical University Hospital, Taipei, Taiwan; 2 Division of Neurosurgery, Department of Surgery, School of Medicine, College of Medicine, Taipei Medical University, Taipei, 11031 Taiwan; 3 Taipei Neuroscience Institute, Taipei Medical University, Taipei, Taiwan

Correction to: *Scientific Reports* 10.1038/s41598-020-62869-0, published online 07 April 2020

The original version of this Article contained an error in Affiliation 2, which was incorrectly given as ‘Department of Surgery, School of Medicine, Taipei Medical University, Taipei, Taiwan’. The correct affiliation is listed below:

Division of Neurosurgery, Department of Surgery, School of Medicine, College of Medicine, Taipei Medical University, Taipei 11031, Taiwan

The original Article has been corrected.

