# Supplementary material for: Teriparatide Associated with Fewer Refractures and Higher Body Heights of Cemented Vertebrae after Vertebroplasty: A Matched Cohort Study
Source: Sci Rep. 2020 Apr 7;10:6005. doi: 10.1038/s41598-020-62869-0 (PMC7138790; doi:10.1038/s41598-020-62869-0)
Supplement: Supplementary file 1 — Supplementary table 1. [file 41598_2020_62869_MOESM1_ESM.pdf]

Teriparatide Associated with Fewer Refractures and Higher Body Heights of Cemented Vertebrae after Vertebroplasty: A Matched Cohort Study

Yi-Shang Yang[1,3*], Yi-Syue Tsou[1,3*], Wen-Cheng Lo[1,2,3], Yung-Hsiao Chiang[1,2,3], Jiann-Her Lin[1,2,3#]

1. Department of Neurosurgery, Taipei Medical University Hospital, Taipei, Taiwan

2. Department of Surgery, School of Medicine, Taipei Medical University, Taipei, Taiwan

3. Taipei Neuroscience Institute, Taipei Medical University, Taipei, Taiwan

* Yi-Syue Tsou and Yi-Shang Yang contributed equally to this work.

# Corresponding author:

Jiann-Her Lin

Department of Neurosurgery, Taipei Medical University Hospital, Taipei, Taiwan

Division of Neurosurgery, Department of Surgery, School of Medicine, College of Medicine, Taipei Medical University, Taipei, Taiwan

Address: No. 252 Wu-Shing street, Taipei, Taiwan

E-mail: jiannher@me.com

Telephone: +886-970405133

Supplement Table 1 Demographics of total VP and TP groups

	Total VP	TP	*P-Value*
n	133	21	
Fractures	167	35	
Age	75.67 ± 9.11	79.19 ± 7.08	0.08
Gender			
F	109	17	0.945
M	24	4	
BMI			
	24.44 ± 3.9	23.14 ± 4.46	0.096
BMD			
	-1.91 ± 1.32	-2.68 ± 0.98	0.005**
Level			
T6	1	0	
T7	4	1	
T8	4	4	
T9	7	2	
T10	6	2	
T11	17	1	
T12	42	7	
L1	42	6	
L2	18	7	
L3	15	3	
L4	7	1	
L5	4	1	

BMD: bone marrow density; BMI: body mass index; TP: teriparatide; VP: vertebroplasty
